# Critical inhaler errors in asthma and COPD: a systematic review of impact on health outcomes

**DOI:** 10.1186/s12931-017-0710-y

**Published:** 2018-01-16

**Authors:** Omar Sharif Usmani, Federico Lavorini, Jonathan Marshall, William Christopher Nigel Dunlop, Louise Heron, Emily Farrington, Richard Dekhuijzen

**Affiliations:** 1 Airway Disease, NHLI, Imperial College London & Royal Brompton Hospital, Dovehouse Street, London, SW3 6LY UK; 2 Department of Experimental and Clinical Medicine, Careggi University Hospital, Florence, Italy; 3 Mundipharma International Limited, Cambridge Science Park, Cambridge, CB4 0AB UK; 4 Adelphi Values, Adelphi Mill, Macclesfield, Cheshire, SK10 5JB UK; 5 Radboud University Medical Center, Nijmegen, Netherlands

**Keywords:** Obstructive lung diseases, Adherence, Errors, Aerosols, Inhalers

## Abstract

**Background:**

Inhaled drug delivery is the cornerstone treatment for asthma and chronic obstructive pulmonary disease (COPD). However, use of inhaler devices can be challenging, potentially leading to critical errors in handling that can significantly reduce drug delivery to the lungs and effectiveness of treatment.

**Methods:**

A systematic review was conducted to define ‘critical’ errors and their impact on health outcomes and resource use between 2004 and 2016, using key search terms for inhaler errors in asthma and COPD (Search-1) and associated health-economic and patient burden (Search-2).

**Results:**

Search-1 identified 62 manuscripts, 47 abstracts, and 5 conference proceedings (*n* = 114 total). Search-2 identified 9 studies. We observed 299 descriptions of critical error. Age, education status, previous inhaler instruction, comorbidities and socioeconomic status were associated with worse handling error frequency. A significant association was found between inhaler errors and poor disease outcomes (exacerbations), and greater health-economic burden.

**Conclusions:**

We have shown wide variations in how critical errors are defined, and the evidence shows an important association between inhaler errors and worsened health outcomes. Given the negative impact diminished disease outcomes impose on resource use, our findings highlight the importance of achieving optimal inhaler technique, and a need for a consensus on defining critical and non-critical errors.

**Electronic supplementary material:**

The online version of this article (10.1186/s12931-017-0710-y) contains supplementary material, which is available to authorized users.

## Background

Inhaled drug delivery is the cornerstone of therapy for the treatment of obstructive chronic airway diseases, such as asthma and chronic obstructive pulmonary disease (COPD) [1]. The most common devices used to administer aerosolized medication in day-to-day respiratory practice are the pressurized metered-dose inhaler (pMDI) and the dry powder inhaler (DPI). pMDIs are most often prescribed [2], but patients need to inhale correctly and coordinate breathing and actuation to ensure effective drug delivery [3–6]. In contrast, DPIs are breath-actuated, with most devices relying on a rapid and powerful inhalation manoeuvre for drug delivery, which can be particularly problematic for patients who struggle to inhale forcefully [6].

Recent advances in inhaler technologies have seen an explosion in the number of devices [7]. This plethora of devices, however, has led to confusion in their use amongst health-care providers (HCPs) and patients, who may not properly understand how to use inhalers [8]. Indeed, mastering an inhaler device involves correct preparation and handling of the device before inhalation, and an optimal inhalation technique; an error in any step of this process may lead to inadequate drug delivery to the lungs.

There is no one ‘perfect device’ and several studies have shown that inhaler technique errors made by patients with asthma and COPD are common in real life with both pMDIs and DPIs despite advances in inhaler device technology [3, 9–12]. Although study results vary, estimates of those making inhaler errors range up to 90% of patients irrespective of the device type used [13, 14]. Most importantly, it is vital to distinguish between ‘critical’ (sometimes defined as ‘essential’ or ‘crucial’) errors, which are likely to significantly impair the delivery of adequate medication to the lungs, and ‘non-critical’ errors, which are likely to result in a reduced amount of drug reaching the lungs compared with that attained using the correct technique [15, 16].

A recent major cross-sectional study of asthma patients has compared inhaler technique data with data on disease control, in order to determine which errors are most associated with poor health outcomes [17]. The results of this may provide the most coherent basis for defining and identifying critical errors; however, progress towards fully elucidating these errors is slow.

The societal and health-economic burden of poor inhaler technique is increasingly being recognised [10]. Worryingly, in three countries (the UK, Spain and Sweden) poor inhaler technique accounted for over €750 million in direct and indirect costs in 2015, for the two most commonly used DPIs [18]. These cost data, together with the increasing prevalence of obstructive lung diseases and restriction in healthcare spending is propagating the imperative need for inhaler competency (that is, correct and effective inhaler use) [15].

Recent global position documents from the Global Initiative for Asthma (GINA) and Global Initiative for Chronic Obstructive Lung Disease (GOLD) both give significant prominence to assessing and correcting poor inhalation technique before escalating drug therapy [19, 20].

Price et al. proposed the need for policy change and research focusing on current gaps in knowledge: specifically on the association between device errors and health economic and clinical outcomes, and on the patient characteristics associated with a higher frequency of errors [15]. Indeed, clinicians must recognise that the device itself and its characteristics are at least equally as important as the prescribed drug; and that in future, the choice of drug compound may be considered to be of secondary importance [3].

The aim of this study was to define ‘critical’ errors and their impact on health outcomes and resource use between 2004 and 2016. This was accomplished through systematically reviewing the scientific literature on inhaler errors made by patients when using pMDIs and DPIs, and the approaches used to assess them -- exploring the relationships between inhaler errors, disease outcomes, quality of life, and healthcare resource use, and associations between patient characteristics and inhaler errors. Given the striking variety of inhaler errors reported in the literature [11], this paper focuses on critical errors, as these are most likely to have a health impact.

## Methods

### Overview

This systematic review was undertaken in accordance with the methodological and reporting standards recommended by PRISMA [21], and was registered in the PROSPERO international prospective register of systematic reviews (CRD42016036118). The review consisted of two distinct searches: search-1 focused on definitions and descriptions of critical errors, and search-2 aimed to identify the literature regarding economic models on the cost of critical errors and patient burden (see Additional file 1: Table S1).

### Inclusion criteria

Studies from search-1 were included if they reported data on inhaler errors with pMDI and/or DPIs in patients with asthma or COPD, and if they related inhaler technique to disease outcomes or quality of life (QoL). Studies from search-2 were included if they reported data on the patient and/or economic burden of inhaler errors. Soft-mist inhalers and nebulisers were not considered in either search, as pMDIs and DPIs are estimated to make up around 99.8% of the global market share of inhaler devices [22].

Both searches were conducted, reviewed, and each article checked, by two authors (LH, EF) in four online databases (Embase, Medline, EconLIT and Evidence-Based Medicine Reviews), limited to studies published in English between 2004 and May 2016. International conference proceedings from 2013 to 2016 were also scanned (see Additional file 1: Table S1). All the authors reviewed the finalized list of selected articles for approval.

### Data extraction

The following data were recorded from each selected article: author and contact details; number of patients; patient characteristics including age, gender, education, comorbidities, socioeconomic class, concurrent device use, and previous instruction; type(s) of inhaler; type(s) of inhaler error(s); definition of critical error(s); type of disease (asthma, COPD, or both); and findings on disease outcomes or QoL.

We grouped the emergent themes into 5 domains in our systematic review; (1) patient characteristics, (2) educational aspects, (3) disease outcomes, (4) quality of life, (5) health economics. This qualitative assessment of the study results allowed results to be reported more clearly, in order to help explore the impact of critical errors on health outcomes and resource use.

When studies were examined for evidence of an association between patient characteristics and presence or rate of inhaler errors, an a priori predefined list of characteristics agreed by consensus between the authors was used to focus analysis. This included: patient age, gender, socioeconomic class, education level, inhaler education, comorbidities, and the number of inhaler devices prescribed at the same time.

Additionally, the reference lists of all retrieved papers were reviewed for any potentially relevant studies, and editorials, commentaries, case studies, letters and opinion pieces were excluded. Studies examining nebuliser inhaler errors or those pooling nebulizer inhaler errors data with data for other inhalers were excluded, as our aim was to assess inhaler devices that administer a single discrete dose. Studies that did not specify inhaler types were included, as it was considered likely that pMDIs and DPIs would have been used, due to these of inhaler types comprising the majority of market share [23]. Descriptive methods were used to analyse data for the associations mentioned above.

## Results

### Search results

**Fig. 1:**
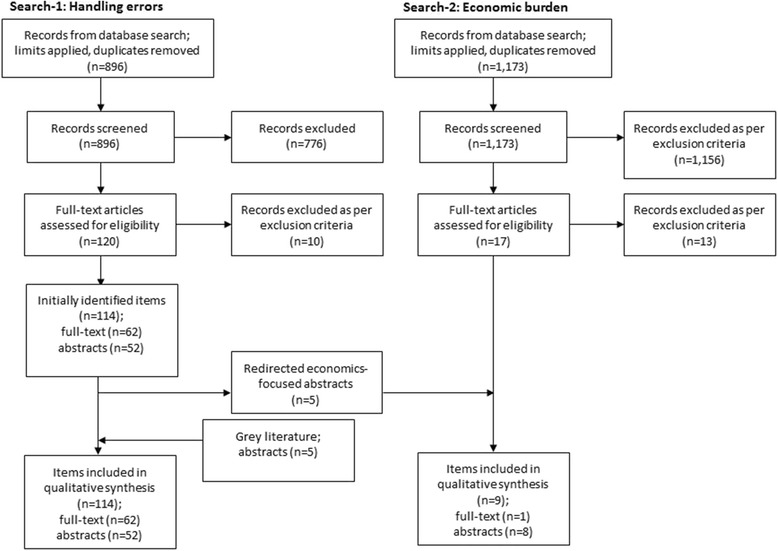
PRISMA diagram. The database search and analysis in Search-1 initially yielded 114 full-text articles or abstracts; give abstracts were removed and incorporated into the results of Search-2 due to being economics-focused; a further five abstracts were added to Search-1 following the grey literature search. Therefore, the final yield of Search-1 was 114

Initially (not including grey literature), Search-1 yielded 114 studies: 62 of these were full-text articles and 52 were abstracts. Of these, five abstracts were identified as having an economics focus and were therefore moved to the results of Search-2 (Fig. 1). Following the addition of five grey literature abstracts, the total yield of Search-1 was again 114 studies.

**Fig. 2:**
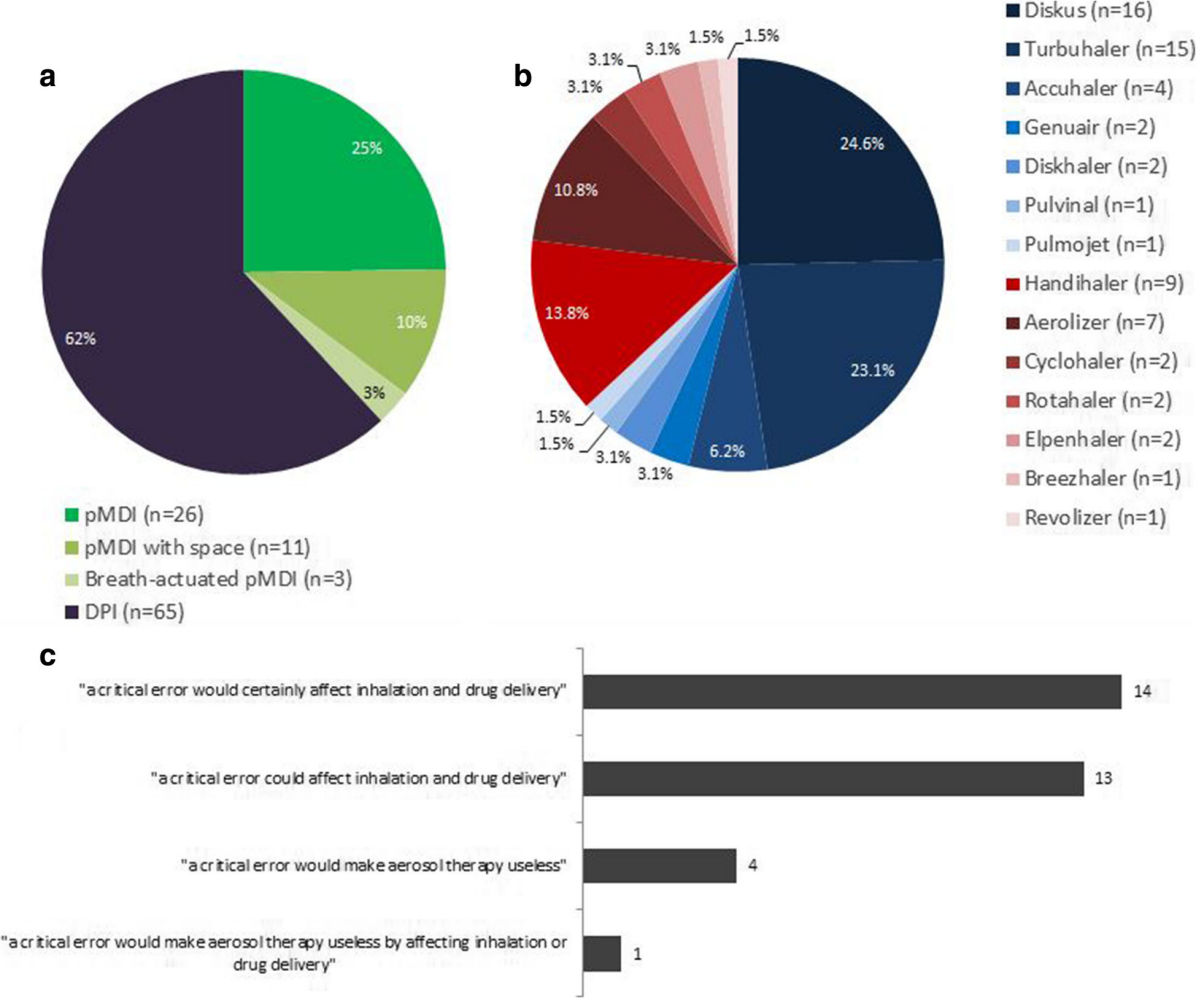
**a**. Journal articles reporting critical inhaler errors for pMDIs and DPIs. Note: percentages are calculated as a proportion of total mentions (*n* = 105) of each inhaler type by all inhaler error studies. Individual studies may mention more than one inhaler type. **b**. Journal articles reporting critical inhaler errors for specific DPI device types, both multi-dose (blue) and single-dose (red). Note: percentages are calculated as a proportion of total mentions (*n* = 65) of all device types by all inhaler error studies. Individual studies may mention more than one inhaler type. **c**. Studies stating a definition of a critical error, separated into categories. Details of each study and the exact wording used by each are presented in Additional file 1: Table S4. Note: In this figure the term “critical error” refers to both critical errors and critical steps that, when performed incorrectly, constitute critical errors

All studies (*n* = 114) in Search-1 reported inhaler error data on pMDIs with or without spacers, and single- or multiple-dose DPIs (Fig. 2a and b). Study details including population age, respiratory disease, and inhaler device type are presented in Table 1.

**Table 1:** Journal articles (*n* = 63) and abstracts (*n* = 60) identified within the literature search

Study details				Content details			Significant association of patient characteristics with errors						
Author	Disease	Inhaler types	n	Disease outcomes	Quality of life	Economic models	Age	Gender	Education	Previous instruction	Comorbidities	Socioeconomic class	Concurrent device use
Al Doghim [113]	A	p, d	56	–	–	–	–	–	–	–	–	–	–
Al Hassan et al. [62]	C	p	100	–	–	–	×	–	✓	×	–	–	–
Al Jahdali et al. [67]	A	p, p + s, d	450	✓	–	–	×	×	×	✓^h^	–	–	–
Al Somali et al. [115]	C	d	24	–	–	–	–	–	–	–	–	–	–
Al Zabadi and El Sharif. [152]	A	u	121	–	–	–	–	–	–	–	–	–	–
Ammari et al. [111]	A	p	30	✓^a^	–	–	–	–	–	–	–	–	–
Azouz et al. [153]	AC	p	111	×^g^	–	–	–	–	–	–	–	–	–
Baddar and Rawas. [154]	A	u	218	–	–	–	–	–	–	–	–	–	–
Baddar et al. [78]	A	u	218	✓	–	–	–	×	–	–	–	–	–
Barnestein-Fonseca et al. [106]	C	u	220	–	–	–	–	–	–	–	–	–	–
Barthwal et al. [83]	A	p, p + s, d	172	✓^a^	–	–	–	–	–	–	–	–	–
Basheti et al. [84]	A	d	97	✓^a^	✓^a^	–	–	–	–	–	–	–	–
Basheti et al. [24]	A	d	186	–	–	–	–	–	–	–	–	–	–
Batterink et al. [25]	C	p, p + s, d	37	–	–	–	–	–	–	×	–	–	✓
Bell et al. [99]	AC	p, pa	NR	–	–	–	–	–	–	–	–	–	–
Benjamin et al. [116]	AC	u	n/a	✓ ^b^	–	–	–	–	–	–	–	–	–
Bijos et al. [129]	AC	u	207,801^q^	–	–	✓	–	–	–	–	–	–	–
Bilal et al. [155]	A	u	NR	–	–	–	–	–	–	–	–	–	–
Bryant et al. [55]	AC	p, p + s, d	103	–	–	–	×	×	–	–	–	-^k^	–
Burkhart et al. [85]	A	p	42	–	–	–	–	–	–	–	–	–	–
Buset et al. [71]	AC	u	43	–	–	–	×	–	–	×	×	–	×
Caliskaner et al. [156]	AC	u	U	–	–	–	–	–	–	–	–	–	–
Camilleri et al. [80]	AC	p,d	167	–	–	–	–	✓	✓	✓	✓	–	–
Capanoglu et al. [53]	A	p + s, d	171	✓	–	–	✓	✓	✓	✓	–	–	–
Capstick et al. [31]	A	u	25	–	–	–	–	–	–	–	–	–	–
Carpenter et al. [86]	A	p, p + s	91	×	–	–	–	–	–	–	–	–	–
Cayo-Quiñe et al. [72]	AC	p	378	–	–	–	✓	–	–	×	–	–	–
Chorão et al. [60]	AC	p, pa, d, s	301	–	–	–	✓	✓	✓^m^	–	–	–	×
Chrystyn et al. [157]	C	d	127	–	–	–	–	–	–	–	–	–	–
Chrystyn et al. [37]	AC	d	421	–	–	–	–	–	–	–	–	–	–
Coelho et al. [38]	A	p, p + s, d	467	✓	–	–	×	×	×	–	–	–	–
Dalcin et al. [56]	A	p, d	268	✓	–	–	×	×	✓^m^	–	✓	✓	–
Deering et al. [158]	C	u	24	–	–	–	–	–	–	–	–	–	–
Deerojanawong et al. [26]	A	p, p + s	93	–	–	–	✓^l^	–	–	✓^i^	–	–	–
Elgendy et al. [95]	A	p	491	–	–	–	–	–	–	–	–	–	–
Estrada et al. [135]	C	u	108	–	–	×^n^	–	–	–	–	–	–	–
Fernandes et al. [105]	A	u	89	✓ ^b^	–	–	–	–	–	–	–	–	–
Ganguly et al. [81]	AC	p, p + s, d	105	–	–	–	–	–	×	×	–	×	–
Garcia-Cardenas et al. [87]	A	d	336	✓^a^	–	–	–	–	–	–	–	–	–
Giraud et al. [39]	A	p, pa	727	✓^a^	–	–	–	–	–	✓	–	–	–
Giraud et al. [57]	A	pa	6512	✓	–	–	✓	–	–	–	–	–	–
Göris et al. [88]	C	p, d	69	✓^a^	✓^a^	–	–	–	–	–	–	–	–
Groot et al. [125]	A	u	142	×^g^	–	–	–	–	–	–	–	–	–
Grover et al. [96]	A	p + s, d	40	✓ ^a^	–	–	–	–	–	–	–	–	–
Hagmolen et al. [40]	A	p, p + s, d	530	×	–	–	×	×	–	×	–	–	–
Hämmerlein et al. [61]	AC	p, p + s, pa, d	757	–	–	–	×	×	×	✓	–	–	–
Harnett et al. [58]	A	p, d, s	46	×	-^d^	–	×	–	–	–	–	–	–
Hass et al. [159]	A	d, s	48	–	–	–	–	–	–	–	–	–	–
Herscher et al. [160]	A	p,d	452	–	–	–	–	–	–	–	–	–	–
Hesselink et al. [89]	AC	u	276	×	×^c^	–	–	–	–	–	–	–	–
Khan et al. [114]	AC	d	50	–	–	–	–	–	–	–	–	–	–
Khassawneh et al. [82]	AC	p, d	300	–	–	–	−^f^	−^f^	−^f^	–	–	–	✓
Komase et al. [63]	C	d	150	–	–	–	×	×	–	–	–	–	–
Kuna et al. [97]	A	p	16,844	✓	–	–	–	–	–	–	–	–	–
Kuprys-Lipinska et al. [100]	AC	d	7400	×^p^	–	–	–	–	–	–	–	–	–
Lee et al. [66]	A	p, d	360	–	–	–	✓	✓^m^	✓^m^	✓	–	–	–
Lee et al. [110]	A	u	92	–	–	–	–	–	–	–	–	–	–
Leiva-Fernandez et al. [161]	C	p, d	465	–	–	–	–	–	–	–	–	–	–
Levy et al. [90]	A	p, p + s, pa	3981	✓	–	–	–	–	–	–	–	–	–
Lewis et al. [131]	AC	u	1.3 m^q^	–	–	✓	–	–	–	–	–	–	–
Lewis et al. [132]	AC	d	266,657^q^	–	–	✓	–	–	–	–	–	–	–
Lewis et al. [133]	AC	d	167,666^q^	–	–	✓	–	–	–	–	–	–	–
Lewis et al. [134]	AC	d	409,445^q^	–	–	✓	–	–	–	–	–	–	–
Li et al. [51]	C	d	384	–	–	–	–	–	–	–	–	–	–
Lin et al. [101]	A	u	32	–	–	–	–	–	–	–	–	–	–
Loh et al. [59]	A	p	134	×	–	–	×	×	×	–	–	–	–
Maazuddin et al. [91]	AC	p, p + s, d	104	–	✓^a^	–	–	–	–	–	–	–	–
Madkour et al. [35]	AC	p,d	533	–	–	–	–	–	–	–	–	–	–
Manriquez et al. [69]	A	p + s	263	–	–	–	✓^e^	–	–	–	–	–	–
Maricoto et al. [68]	AC	p, p + s, d	62	✓ ^j^	–	–	×	–	–	✓	–	–	–
Maricoto et al. [104]	AC	u	44	✓^j^	–	–	–	–	–	–	–	–	–
Mazankova et al. [162]	A	p, pa, d	200	–	–	–	–	–	–	–	–	–	–
Mehuys et al. [41]	C	p, p + s, pa, d	555	–	−^d^	–	–	–	–	–	–	–	–
Melani et al. [10]	AC	p, d	1664	✓	–	–	✓	×	✓	✓	–	–	×
Melani et al. [52]	AC	p, d	1527	–	–	–	–	–	–	–	–	–	–
Minai et al. [92]	A	p	45	–	–	–	–	–	–	–	–	–	–
Molimard et al. [42]	A	p, d	4362	×^g^	–	–	–	–	–	–	–	–	–
Molimard et al. [47]	–	p, pa, d	3811	–	–	–	–	–	–	–	–	–	–
Muhammad et al. [163]	A	p, p + s	181	–	–	–	–	–	–	–	–	–	–
Nama et al. [32]	C	u	46	–	–	–	–	–	–	–	–	–	–
Nolan-Neylan et al. [65]	C	d	150	–	–	–	×	×	×	–	–	–	×
Öztürk et al. [36]	C	p,d	180	–	–	–	–	–	–	✓	–	–	–
Öztürk et al. [64]	C	d	442	–	–	–	✓^e^	–	×	–	–	–	–
Pascual et al. [54]	C	d	128	–	–	–	–	–	–	–	–	–	–
Plaza et al. [98]	A	u	230	✓ ^b^	✓^b^	–	–	–	–	–	–	–	–
Pothirat et al. [34]	C	p, p + s, d	103	×	×	–	×	×	✓	–	–	–	×
Prieto-Centurion et al. [108]	C	p,d	31	–	–	–	–	–	–	–	–	–	–
Rajan et al. [43]	AC	d	100	–	–	–	–	–	–	–	–	–	–
Roggeri et al. [128]	AC	.	200^q^	–	–	✓^o^	–	–	–	–	–	–	–
Roggeri et al. [127]	AC	u	200^q^	–	–	✓^o^	–	–	–	–	–	–	–
Ronk et al. [112]	A	p	45	–	–	–	–	–	–	–	–	–	–
Rootmensen et al. [44]	AC	p, p + s, d	156	–	–	–	✓^m^	×	×	✓	–	–	✓
Sadowski et al. [79]	AC	p, p + s, d	161	–	–	–	–	✓	–	–	✓	–	–
Sangita et al. [73]	C	p,d	100	–	–	–	✓^i^	×	✓^i^	–	–	–	–
Santos et al. [164]	AC	u	50	–	–	–	–	–	–	–	–	–	–
Schulte et al. [45]	AC	d	72	–	–	–	–	–	–	–	–	–	–
Shetty et al. [102]	A	p	35	–	–	–	–	–	–	–	–	–	–
Souza et al. [165]	AC	p, d	120	–	–	–	–	–	–	–	–	–	–
Sriram et al. [70]	C	u	150	×	–	–	×	×	×	–	–	–	×
Sulaiman et al. [117]	A	u	221	✓ ^b^	–	–	–	–	–	–	–	–	–
Takemura et al. [103]	A	u	289	–	×	–	–	–	–	–	–	–	–
Tarsin et al. [166]	AC	p, d	300	–	–	–	–	–	–	–	–	–	–
Thomas et al. [118]	A	p, d	162	–	–	–	–	–	–	–	–	–	–
Thomas et al. [50]	A	d	60	–	–	–	–	–	–	–	–	–	–
Torvinen et al. [130]	AC	d	n/a	–	–	✓	–	–	–	–	–	–	–
Turan et al. [76]	C	u	76	–	–	–	×	×	×	✓	×	–	–
Udwadia et al. [75]	A	p	241	–	–	–	×^n^	–	×^n^	–	–	–	–
van der Palen et al. [119]	C	p, d	567	–	–	–	–	–	–	–	–	–	–
van der Palen et al. [27]	AC	d	113	–	–	–	–	–	–	–	–	–	–
van der Palen et al. [28]	C	d	130	–	–	–	–	–	–	–	–	–	–
van der Palen et al. [46]	C	d	105	–	–	–	–	–	–	–	–	–	–
van der Palen et al. [93]	C	d	60	–	–	–	–	–	–	–	–	–	–
van der Valk et al. [107]	AC	d	113	–	–	–	–	–	–	–	–	–	–
Vanderman et al. [33]	AC	p,d	24	–	–	–	×	–	–	–	×	–	×
Vilamil-Osorio et al. [74]	A	p	118	–	–	–	×	×	×	×	–	–	–
Voshaar et al. [29]	A	d	66	–	–	–	–	–	–	–	–	–	–
Westerik et al. [48]	A	d	623	✓	–	–	×	✓	✓^m^	✓	✓	–	–
Wiacek et al. [109]	AC	d	7100	×^p^	–	–	–	–	–	–	–	–	–
Wieshammer et al. [30]	AC	d	224	–	–	–	✓	×	–	✓^i^	–	–	×
Williams et al. [167]	AC	p, p + s, d	59	–	–	–	–	–	–	–	–	–	–
Wu et al. [77]	AC	p, d	35	–	–	–	×	×	–	–	×	–	–
Yildiz et al. [94]	A	p, d	572	✓^a^	–	–	–	–	–	–	–	–	–
Zaidi et al. [126]	AC	p, p + s, d	59	×	–	–	–	–	–	–	–	–	–

Key: *A* Asthma, *C* COPD, *AC* Asthma & COPD; *p* pMDI, *p + s* pMDI with spacer, *pa* Breath actuated pMDI, *d* DPI, *s* Soft mist inhaler, *u* Unspecified, ✓ = significant, × = non-significant

Note: ^a^Significant improvement observed following inhaler training interventions which improved inhaler technique. ^b^Significant improvements in disease outcomes or QoL, or reduction in hospital admissions reported following inhaler technique intervention but did not collect inhaler error rates. ^c^No significant impact on QoL was reported from an intervention that significantly improved inhaler technique. ^d^QoL impairment reported in asthma or COPD not related to inhaler errors. ^e^Age associated only with some error types. ^f^Data on factors potentially associated with error rate was recorded, but findings were not reported. ^g^Improvements reported but statistical analysis was not provided. ^h^Patients who had received “education about medication” were more likely to use their inhaler properly, relative to patients who had not; however, it is not explicitly stated that this education included inhaler technique instruction. ^i^Dependent on device type. ^j^Significant improvements reported in asthma but not COPD. ^k^Socioeconomic class was examined by proxy; however, no data or findings are presented on this factor or its association with errors. ^l^Data was not provided on all device types. ^m^Significant after univariate analysis only. ^n^Statistical analysis was not provided. ^o^The two items published by Roggeri et al. are suspected to report the same economic model, and are therefore counted as one significant result in this analysis. ^p^The two items published by Kuprys-Lipinska et al. and Wiacek et al. are suspected to report the same study, and are therefore counted as one negative result in the disease outcomes analysis; also, improvements in disease control were reported but statistical analysis was not provided. ^q^Population numbers reported for economic modelling studies marked with this symbol reflect the hypothetical patient population within the model

Search-2 on the health-economic burden of inhaler errors yielded only one full-text article and three abstracts that fulfilled the inclusion criteria, to which five abstracts from Search-1 were added (Fig. 1).

### Definition of critical inhaler errors

Among the 36 studies giving specific examples of ‘critical’ errors, 32 included a definition of ‘critical’ inhaler errors, and the definition itself substantially varied between the studies (Fig. 2c). In most cases, studies did not provide information on the origin of their definition of a critical error; however, where this information was provided, definitions were commonly taken from previous studies, rather than being formulated by the study researchers. Astonishingly, our search yielded 299 descriptions of critical errors across the device types.

The most common definition was an action affecting the lung deposition of inhaled drug, resulting in little or no medicine being inhaled or reaching the lungs (*n* = 27), where 14 definitions stated a critical error “would” certainly affect inhalation and drug delivery [24–37], and 13 others said a critical error “could” affect these [38–50]. Conversely, 4 papers defined a critical error in terms of effectiveness: that is, an error that would make aerosol therapy useless [10, 51–53]; and Pascual used a combined definition of deposition and effectiveness: that is, “an error that compromised the potential benefit of the treatment, such as impeding drug deposition or the delivery of an insufficient dose” [54].

Surprisingly, only sixty studies (53%) used a checklist to quantify errors and to enable comparisons between devices. However, these checklists were often created by the authors themselves (either taken from previous studies, or were copied from the instructions provided with the inhaler device), without external validation of the checklist itself for each device type. The number of critical errors described varied by device type and by study (Additional file 1: Table S2).

To further compound matters, there were also differences in the descriptions of the actual errors themselves. For example, one DPI error was described in four different ways: two studies mentioned the critical steps which, if not performed, would be errors: “slide lever as far as possible” [42]; “push lever back completely” [27]; and two gave differing terminology for the critical error: “failure to slide the lever until the ‘click’ sound” [51] and “not sliding back the lever until a click is heard” [30]. Similarly, there were also differences in agreement between the authors of the different studies in the categorization of a critical error versus a non-critical error, once again affecting attempts to compare studies and collectively understand the impact of inhaler errors in daily clinical practice. For example, not holding the inhaler upright whilst using a pMDI was referred to as a critical error or step by three studies [39, 42, 44], but Bryant defined it simply as an “error” [55].

While many studies reported associations between characteristics or patient experiences and errors, these did not specify whether associations existed with critical errors specifically, or with all errors.

#### Effects of patient characteristics on frequency of inhaler errors

Overall, 41 studies of 114 (36%) investigated the effect of predefined patient characteristics on inhaler error frequency (Table 1), with patient age, gender, level of education, number of devices prescribed, and previous inhaler instruction being the most commonly explored factors.

Of 33 studies which examined the effect of patient age [10, 26, 30, 33, 34, 38, 40, 44, 48, 53, 55–77], 29 were in adults and 4 were paediatric. Only twelve studies (36%) reported age to be significantly associated with worsening frequency of inhaler errors [10, 26, 30, 44, 53, 57, 60, 64, 66, 69, 72, 73], whereas 21 studies found no significant association. In 7 studies, older adults were found to make significantly more errors [10, 30, 44, 57, 60, 64, 66]. Of the 4 paediatric studies [26, 40, 53, 74], two reported a significant association between age and frequency of errors: Deerojanawong reported that younger children made errors significantly more frequently [26], while Capanoglu reported the opposite finding: that older children made errors more frequently [53]. Twenty-five studies of 114 (22%) reported on the effects of gender [10, 30, 34, 38, 40, 44, 48, 53, 55, 56, 59–61, 63, 65–67, 70, 73, 74, 76–80], where 6 studies concluded a significant impact on inhaler error frequency, but the results were contradictory and inconclusive as to whether male or female gender was associated with poor technique [48, 53, 60, 66, 79, 80]. Of the 22 studies that reported a trend between low education and high inhaler error frequency [10, 34, 38, 44, 48, 53, 56, 59–62, 64–67, 70, 73–76, 80, 81], 10 found the association to be statistically significant [10, 34, 48, 53, 56, 60, 62, 66, 73, 80].

Of the 114 articles, 21 studies (18%) explored the relationship between previous inhaler instruction and inhaler error frequency [10, 25, 26, 30, 36, 39, 40, 44, 48, 53, 61, 62, 66–68, 71, 72, 74, 76, 80, 81], where 11 studies found previous education or instruction to be significantly related to better inhaler technique [10, 36, 39, 44, 48, 53, 61, 66, 68, 76, 80]. One study by Al-Jahdali reported a significant relationship between ‘lack of education about medication’ and improper device use [67], and two reported that reduced error frequency among previously instructed patients was dependent on device type, where technique improved only in patients using MDI spacer [26] or Diskus or Turbuhaler. [30] Interestingly, a third of studies (*n* = 7) reported that previous instruction in inhaler use did not significantly affect inhaler technique [25, 40, 62, 71, 72, 74, 81].

Specifically, we noted a statistically significant relationship was reported between increased error frequency and other patient characteristics such as having the presence of two or more comorbidities [56], obesity [48], heart disease [80], cognitive impairment or neuropathy [79], and lower socioeconomic class [56]. There were contradictory results between three studies that reported significant results for whether a higher or lower number of devices prescribed concurrently impacts error frequency [25, 44, 82].

#### Effects of educational intervention on frequency of inhaler errors

Educational interventions and their relationship to inhaler errors were addressed in 52 articles [27–29, 34, 37, 39, 45, 49–51, 54, 57–59, 61, 83–119], but studies varied in how errors were assessed (the tools used), by whom (the healthcare personnel), and in the duration of intervention. Interventions were undertaken by face-to-face consultation with a variety of HCPs involving physicians (*n* = 5), nurses (*n* = 5), paramedics (*n* = 1), and pharmacists (*n* = 8). In other studies, a video (*n* = 5), leaflet instructions (*n* = 17), or an online program were used (*n* = 2). Thirty-two studies undertook patient assessments before and after the educational intervention [29, 34, 39, 45, 51, 57–59, 61, 83–92, 94, 95, 97, 100–104, 108, 109, 114, 115]. Where 26 studies positively reported a significant improvement in inhaler technique following the intervention [29, 34, 39, 51, 57, 58, 61, 83–89, 92, 94, 95, 97, 101, 103, 104, 108, 109, 114, 115].

Analysis of interventions noted that the majority of the pharmacist-led studies, seven of the eight, demonstrated a statistically significant improvement in inhaler technique [39, 61, 84, 87, 91, 101, 103]. Of the five nurse-led interventions [34, 58, 85, 90, 99], three succeeded in significantly improving inhaler technique [34, 58, 85], and two reported a decrease in inhaler error frequency but did not include a statistical analysis [90, 99]. A further six HCP studies reported statistically significant improvements: three physician-led interventions, a physician-and therapist-led study, GP assistant study and a paramedic-led study all reported statistically significant improvements [57, 83, 89, 92, 94, 97]. Four studies with unspecified instructor types reported improvement [45, 51, 95, 102], but only two provided statistical analysis [51, 95]. Of the leaflet-based intervention studies (*n* = 17) [27–29, 37, 45, 49, 50, 54, 88, 93, 104, 106, 107, 112, 115, 118, 119], five compared inhaler technique before and after the intervention, of which four reported a significant improvement in inhaler technique [29, 88, 104, 115].

#### Association between disease outcomes and inhaler errors

Thirty-six of the 114 studies (Table 1) examined disease outcomes in relation to inhaler errors or inhalation technique (see Table 2 for a summary of available odds ratios). In the assessment of asthma control, the most common measurements were the Asthma Control Questionnaire (ACQ) and Asthma Control Test (ACT) instruments. [120, 121] Other measurements included: the Control of Allergic Rhinitis and Asthma Test [122], Asthma Therapy Assessment Questionnaire [123], Test For Respiratory And Asthma Control In Kids scales [124], frequency of exacerbations, emergency healthcare use, or general classification of patients into levels of disease control using the Global Initiative for Asthma (GINA) criteria. For COPD patients, disease outcomes were measured by: Baseline Dyspnoea Index (BDI), rates of exacerbations, hospitalizations, or by degree of dyspnoea using the modified Medical Research Council (MRC) questionnaire.

**Table 2:**
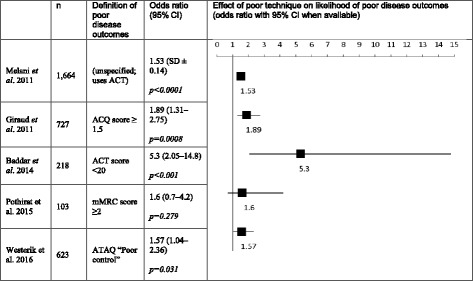
Published odds ratios for baseline associations between poor inhaler technique and poor disease control

Our systematic analysis revealed 10 studies observed a higher inhaler error frequency was significantly associated with poor disease outcomes, primarily in asthma (*n* = 9), but also in asthma and COPD (*n* = 1) [10, 38, 48, 53, 56, 57, 67, 78, 90, 97, 117]. Molimard reported that using a device incorrectly, irrespective of the type, was associated with an increased Asthma Control Score [42], and in another study by Kuprys-Lipinska and Wiacek, over 94% of patients reported an association between improved DPI technique and better disease outcomes in asthma and COPD; however, no statistical analysis was provided [100, 109]. Groot reported that incorrect inhaler technique with unspecified inhalers was the underlying cause of poor asthma control in 7.8% of its population, but again did not provide statistical analysis [125].

We identified eight studies where the inhaler training interventions (including such aspects as physical demonstration, technique labels, and written action plans) led to an improvement in inhaler technique and also a significant increase in disease control, (seven in asthma [39, 83, 84, 87, 94, 96, 111], and one in COPD [88]) while 4 studies reported that their intervention significantly improved disease outcomes or reduced hospital admission frequency, but did not measure inhaler error frequency [98, 105, 116, 117]. Two further studies reported that training significantly improved technique and outcomes in asthma, but not in COPD patients [68, 104]. Eight studies reported no significant relationship between poor inhaler technique or errors with asthma control [34, 40, 58, 59, 70, 86, 89, 126].

#### Association between quality of life and inhaler errors

Seven of the 114 studies examined any association between QoL and inhaler errors: three in asthma, two in COPD and two in a mixed population.

A significant improvement in QoL was reported in three studies, following interventions which improved inhalation technique [84, 88, 91]. Basheti observed a significant correlation between improvement in DPI technique and improvement in asthma-related QoL following a pharmacist-led training intervention [84]. Goris reported significant improvements in QoL according to the St. George’s Respiratory Questionnaire (SGRQ) in all domains of QoL following intervention (aided by a movie and leaflet) in pMDI and DPI technique in COPD patients [88]. Maazuddin reported a pharmacist-led intervention led to significant improvements in the SGRQ outcomes in patients with COPD for pMDI and aerosol based devices including, *Autohaler*® and *Evohaler*®, but not for the three DPIs *Revolizer*®, *Rotahaler*® and *Starhaler*® [91].

A further study by Plaza reported a clinically significant increase in Mini Asthma Quality of Life Questionnaire scores among patients receiving a repeated training intervention (including development of a personalised action plan) delivered by a professional educator, physician or nurse, but the inhaler error frequency was not captured [98].

In contrast, an RCT by Hesselink found no significant impact on QoL in asthma and COPD (measured using the Quality-of-Life for Respiratory Illness Questionnaire) following a family practice assistant intervention (involving a structured consultation and use of checklist), although a significant improvement in inhaler technique was recorded [89]. An observational, retrospective study by Takemura reported unchanged SGRQ scores in asthma patients following intervention at regular intervals of at least 6 months by certified participants in a community-pharmacist educational program [103]. A further study by Pothirat captured a non-significant relationship between inhaler errors and poor quality of life, as judged by the by COPD Assessment Test (CAT) [34].

#### Economic models investigating the costs associated with inhaler errors

Our analysis identified eight studies [127–135], of which one was reported both in manuscript and abstract form [127, 128].

Roggeri reported a modelling study conducted in Italy and calculated the increased healthcare resource use by asthma or COPD patients making one or more critical inhaler error and showed this was associated with an additional yearly cost of €44,104 (asthma) or €23,444 (COPD) per 100 patients [127, 128]. Contextualising this for COPD, 100 patients making at least one inhaler error would require 11.5 additional hospitalisations, 13 emergency room visits, 19.5 courses of antimicrobials, and 47 courses of oral corticosteroids, compared to 100 patients not making any critical errors. Corresponding figures for 100 asthma patients were 19 hospitalisations, 26.5 emergency room visits, 4.5 antimicrobial courses and 21.5 oral corticosteroid courses [127, 128].

Bijos modelled the impact of poor inhaler technique on healthcare resource use in Poland, and concluded that misuse of inhaled corticosteroid and long-acting beta agonist fixed-dose combinations resulted in an annual loss of 378 million PLN (€91.1 million) in direct costs and 20.4 million PLN (€4.9 million) in indirect productivity, costs across asthma and COPD [129].

Torvinen calculated the effect on disease outcomes and the economic impact of a new DPI inhaler reported to reduce inhalation errors through innovative inhaler characteristics, and showed a potential saving of €57.78 million, based on a 10.1% rate of uptake among 701,983 patients with persistent asthma or COPD in Italy when switching to the new device from their existing DPI inhalers of *Turbuhaler*® or *Diskus*®, by year 5 of the model [130].

In the UK, Lewis considered the impact of inhaler errors on the economic burden of asthma and COPD with inhaled corticosteroid/long-acting beta-agonist (ICS/LABA) fixed-dose combinations [131]. The authors estimated that 366,000 of the 1.3 million persistent asthma/COPD patients within the UK have poor inhalation technique, and that this was associated with 11.8% (£16.2 million) of unscheduled health care events per year [131].

In a further study, Lewis estimated the additional resource use due to poor inhalation technique in Spain, and calculated a loss of €11.54 million due to unscheduled healthcare events among 563,562 asthma and COPD patients using *Turbuhaler*® or *Accuhaler*®. [132] Two similar economic models considered the impact of improved inhalation technique in asthma and COPD and in the UK and Sweden, and concluded that improved technique could save £3.5 million in the UK through reducing the number of unscheduled health events (assuming an update of 25% in years 4 and 5 of the model), and SEK285.4 million (€31.2 million) in Sweden by reducing the number of lost working days [133, 134].

Of note, four out of the 9 studies were related to the same device utilised in studies sponsored by the same company within a year of each other using the same health economic model employing a device switch approach to the study design [130, 132–134].

Conversely, a real world study in COPD (*n* = 108) conducted in Colombia reported that making inhaler errors was associated with a minor increase in monthly cost per patient ($146.9, versus $142.2 for other patients) [135].

Interestingly, no economics-focused studies on a US population were captured.

### Generic issues identified

As previously mentioned, several types of inconsistency or heterogeneity between captured studies were seen during the review – each of which made the analysis of the data challenging.

The inconsistency in defining critical errors versus normal errors makes drawing conclusions on associations difficult, as errors were considered critical or non-critical by different researchers using different definitions; this is an important issue identified throughout the systematic review.

Furthermore, as differing checklists were used, containing differing numbers and descriptions of errors, error frequencies are likely not completely comparable between studies. In addition, “poor technique” was defined differently by different researchers, who commonly used differing thresholds for labelling a patient’s technique as incorrect or poor [55, 56].

In addition, although disease outcomes were captured using known measures of control, the variety of different measures used does make it more difficult to draw firm conclusions on the association between error frequency and asthma control.

## Discussion

The aim of our article was to define ‘critical’ inhaler errors and their impact on health outcomes and resource use; and by doing so, to bring to the attention of physicians the importance of the inhaler device in their daily prescribing in the management of patients with asthma and COPD. Indeed, both GINA and GOLD now highlight the critical importance of assessing inhaler technique to guide appropriate inhaler prescribing, with a concerted drive to educate professionals and patients about the real impact of inhaler errors on the patients’ disease control, as well as on the financial economics of societal health.

To our knowledge this is the first formally registered evidence-based systematic review with a priori clearly formulated questions that documents the wide discrepancies within the literature regarding definitions and descriptions of inhaler errors and their classification as either ‘critical’ or ‘non-critical’. Previous reviews such as that by Basheti et al. focusing on inhaler error checklists have approached these issues [136], although in a different context.

Astonishingly, we observed 299 different descriptions of critical inhaler errors. Even for the same inhaler device type, different terminology was used between different study authors to describe the same inhaler error, and this may contribute to the confusion observed in clinical practice with regards to best inhaler practice and the limitations in determining associations with inhaler errors [8]. This heterogeneity and lack of consensus fundamentally hampers the ability to interpret studies with respect to the impact of inhaler errors. Indeed, the different definitions of critical error could be a contributing factor to extremely different conclusions even with the same inhaler device type; as exemplified in the Melani study where MDI users were significantly less likely to commit critical errors relative to DPI users [10], in contrast to the Batterink study where MDI users were most likely to make critical errors [25].

The lack of consensus between researchers extends to the use of differing inhaler technique checklists. As the checklists used are not standardized, even within individual inhaler device types, comparing error rates between or within inhaler device types is unfeasible. Future research can and should adopt more consistent inhaler technique checklists, as the manufacturers’ instructions are available to form a basis for a checklist in almost all cases.

We observed several important factors, including older age, education status, lack of previous inhaler instruction, and lower socioeconomic class, which were all associated with high inhaler error frequency. In addition, inhaler technique interventions were found to decrease error frequency, and have positive impacts on disease and patient outcomes, as has previously been described in the literature by Basheti et al. [137].

However these findings were not reflected in all studies, likely due to differences in study design and populations. For example, both interventional and observational studies were included, there were different inhaler devices included (i.e. pMDI or DPI), and wide ranging population sizes (between 46 and 6512 individuals), thereby limiting our ability to directly compare the results.

Interestingly, a significant association with error frequency was found for some comorbidities that are known to be strongly correlated with age, such as obesity, heart disease, or cognitive impairment [48, 79, 80]; but despite this, only around a third of studies that examined age itself reported a significant association with error frequency.

Our systematic review identified studies showing an association between inhaler errors and poor asthma control and COPD disease stability. This is in line with a recent individual study that has demonstrated that inhaler errors affect drug delivery [138]. Sulaiman showed in a laboratory environment that deliberately making certain inhaler errors led to a reduced amount of drug reaching the bloodstream [138]. However, the limited quantity of disclosed research in this area may suggest that the term “critical” is being overused, with only a weak basis for categorising errors as such.

In a recent real-world study by Molimard in 2935 patients an increased risk of COPD exacerbation among patients who made a critical inhaler error, was confirmed [139]. A further study by Price determined that the error of “insufficient respiratory effort” was associated with increased asthma exacerbation rate, as well as decreased control in general [140].

Importantly, we identified eight economic models which linked inhaler errors to economic burden, of which one study by Roggeri demonstrated a specific link between critical errors and resource use, leading to an excess cost of many thousands of Euros per 100 patients making critical errors [127, 128]. Indeed, recently Lewis and colleagues showed that poor inhalation technique led to approximately ¾ billion euros in direct and indirect costs for just two DPI inhalers used over 1 year [18].

Previous literature has also demonstrated that poor disease outcomes are linked with worsened QoL and increased resource use and economic burden through increased physician consultation time and lost productivity (Additional file 1: Table S3) [141–146]. Therefore, the issue of inhaler errors is important to address due to the downstream effects on patients, healthcare systems and society.

Our findings clearly illustrate inhaler technique can be affected by the level of instruction from HCPs. It is therefore important to interpret clinical trial results with caution, given that their controlled environment (where all patients are instructed in inhaler use) may not be representative of clinical practice in real life. This issue is especially important in the context of different inhaler devices that may have ergonomic designs and functions, as raised by Scichilone et al. in a 2015 review [147]. The key message here is that in day-to-day practice, it may be an efficient strategy to provide patients at higher risk of errors with additional specifically tailored in-depth support with their inhaler use, to ensure they are confident with the correct technique.

Greater attention is clearly needed on the routine review of inhaler technique in the patient population as a whole, as a recent study by Sanchis reported rates of common inhaler errors to be static over a period of several decades [11], and data also show that despite optimally prescribed inhaled therapy, levels of asthma control and COPD disease stability remain poor [18, 145].

In comparison with a previously published systematic review only on DPI inhaler errors, our review encompasses a wider range of device types including the most commonly used inhaler device, the pMDI [14]. Whilst Lavorini et al. included data on critical errors and provided a definition of a critical error, their study focused on the incidence of errors and the possible implications for clinical effectiveness of inhalers [14]. A key strength of our review is that it integrates the link between inhaler errors and disease outcomes and QoL, and provides a systematic overview of how these critical inhaler errors are being assessed and measured.

Direct comparisons and synthesis of the data were challenging due to mixed methodologies (such as observational cross-sectional, or interventional cross-over designs, and designs intended for descriptive or qualitative analysis), different patient populations, and varied endpoints. Yet, despite these differences we observed clear trends in our data. However, due to the vast differences between studies, this review did not examine clinical outcomes by device, but this is an important area for future research.

Furthermore, only a handful of the reviewed studies directly addressed patient outcomes and the economic burden of inhaler errors. Therefore, further research and potential health-economic modelling to understand the relationship between inhaler technique and disease outcomes, and the subsequent impact on societal healthcare systems, is vitally required.

Although the present study shows associations between inhaler errors and patient outcomes through a review of chronic obstructive respiratory diseases as a whole, future research may be able to probe further into the two diseases (asthma and COPD). For example, the generally older age, poor prognosis and comorbidities of COPD patients may influence the degree to which their QoL is increased by improvements in technique and control [148]. The substantially higher prevalence of comorbidities among COPD patients, relative to asthma patients, also likely impacts inhaler technique and patient QoL [148].

With the variety of definitions identified in our review, difficulties arise in determining whether a particular inhaler type is inherently more vulnerable to critical inhaler errors. Consistent use of our proposed definition and categorization by all researchers internationally would transform this area of research and greatly facilitate quantitative and objective comparison between devices, providing a clearer indication of the associated error rates. This would revolutionise everyday clinical practice, where reliable comparisons of error rates would greatly help physicians and aid informed treatment decisions, ensuring the most appropriate device is prescribed for the individual patient with clear implications for personalised patient management. Further research into the association of patient characteristics with error rate could examine “health literacy”, a patient’s insight into their own treatment and health system, and determine if poor knowledge is a risk factor for poor technique [115, 149–151].

It is clear that inhaler errors have an effect on disease outcomes, and ultimately patient outcomes and economic burden. This in turn will have an impact on overall disease management and affect not only patients but also the wider healthcare system. These findings are increasingly important given the plethora of devices available to HCPs and patients, and highlight the importance of inhaler mastery in managing and treating asthma and COPD.

There is increasing evidence to suggest that correct inhaler technique (mastery) is fundamental for effective therapy, and that inhaler device type and mastery play important roles in improving adherence, clinical outcomes, quality of life, and use of healthcare resources. Evidence suggests that prescribers should consider patients’ mastery of technique (or lack thereof) and ease of use before changing the dose of inhaled medications, switching to a different inhaler, or adding other treatments to the regimen of patients with poorly controlled asthma. Recent international asthma guidelines highlight the importance of testing and ensuring mastery, alongside checking adherence, before increasing or changing therapy.

## Conclusions

In conclusion, the multitude of definitions cited within the literature indicates that there is an urgent need for a consensus in the way in which critical (and non-critical) inhaler errors are defined. We propose defining a critical inhaler error as an action or inaction that in itself would have a definite detrimental impact on the delivery of the drug to the lung, in contrast to a non-critical error which we would define as an action or inaction that in combination with other factors may, or may not, contribute to ineffective delivery of the drug to the lung.

We advocate that there is a real need for an independent international panel of inhalation experts to collectively determine, through evidence and consensus, the definitions of critical and non-critical inhaler errors. If done for each device type, this would demystify the current confusion within the respiratory community.

We also propose that future studies classify individual errors into categories such as inhalation manoeuvre, dose preparation, inhaler handling, device-specific or generic, in order to make comparison and analysis simpler in order to ultimately help healthcare professionals help their patients.

## Additional file

Additional file 1: One supplementary file is associated with this manuscript: “Online Resource – Critical handling errors in asthma and COPD: A systematic review of impact on health outcomes”. This contains: search strategies and details of the analysis conducted within this literature review (**Table S1**); a breakdown of grouped critical errors described in the literature for different inhaler device types (**Table S2**); summaries of pre-existing known publications that show associations between poor disease control, economic burden and poor QoL (**Table S3**); definitions of a ‘critical’ error provided by studies captured within the literature review (**Table S4**). This file is named: “Critical handling errors data supplement update v8_0”. (DOCX 588 kb)

## Abbreviations

ACQ: Asthma Control Questionnaire
ACT: Asthma Control Test
ATAQ: Asthma Therapy Assessment Questionnaire
BDI: Baseline Dyspnoea Index
CAT: COPD Assessment Test
CI: Confidence interval
COPD: Chronic obstructive pulmonary disease
DPI: Dry-powder inhaler
GINA: Global Initiative for Asthma
GOLD: Global Initiative for Chronic Obstructive Lung Disease
GP: General Practitioner
HCP: Healthcare provider
ICS: Inhaled corticosteroid
LABA: Long-acting beta agonist
MDI: Metered-dose inhaler
mMRC: Modified Medical Research Council (questionnaire)
pMDI: Pressurized metered-dose inhaler
PRISMA: Preferred Reporting Items for Systematic Reviews and Meta-Analyses
QoL: quality of life
RCT: Randomised controlled trial
SGRQ: St George’s Respiratory Questionnaire
