# Supplementary material for: Critical inhaler errors in asthma and COPD: a systematic review of impact on health outcomes
Source: Respir Res. 2018 Jan 16;19:10. doi: 10.1186/s12931-017-0710-y (PMC5771074; doi:10.1186/s12931-017-0710-y)
Supplement: Additional file 1: — One supplementary file is associated with this manuscript: “Online Resource – Critical handling errors in asthma and COPD: A systematic review of impact on health outcomes”. This contains: search strategies and details of the analysis conducted within this literature review (Table S1); a breakdown of grouped critical errors described in the literature for different inhaler device types (Table S2); summaries of pre-existing known publications that show associations between poor disease control, economic burden and poor QoL (Table S3); definitions of a ‘critical’ error provided by studies captured within the literature review (Table S4). This file is named: “Critical handling errors data supplement update v8_0”. (DOCX 588 kb) [file 12931_2017_710_MOESM1_ESM.docx]

**Additional file**

**Critical inhaler errors in asthma and COPD: A systematic review of impact on health outcomes
AUTHORS:**

Usmani, Omar Sharif^1^ <o.usmani@imperial.ac.uk>

Lavorini, Federico^2^ <federico.lavorini@unifi.it>

Marshall, Jonathan^3^ <jonathan.marshall@mundipharma.com>

Dunlop, William Christopher Nigel^3^ <will.dunlop@mundipharma.com>

Heron, Louise^4^ <louise.heron@adelphivalues.com>

Farrington, Emily^4^ <emily.farrington@adelphivalues.com>

Dekhuijzen, Richard^5^ <richard.dekhuijzen@radboudumc.nl>

**AFFILIATIONS:**

1. Imperial College London and Royal Brompton Hospital, London, SW3 6LY, UK

2. Department of Experimental and Clinical Medicine, Careggi University Hospital Florence Italy

3. Mundipharma International Ltd, Cambridge Science Park, Cambridge CB4 0AB, UK

4. Adelphi Values, Adelphi Mill, Macclesfield, Cheshire SK10 5JB, UK

5. Radboud University Medical Center, Nijmegen, Netherlands

Table S1: Search strategy for Search-1 and Search-2

| **Search one (inhaler errors)** | |
| --- | --- |
| “asthma” OR “chronic obstructive pulmonary disease” OR “COPD” [as multipurpose terms] | |
| AND | "inhal$ technique" OR "inhal$ competence" OR "handling error$" OR "improper use" OR “critical error$” OR “misuse” OR “inhal$ mistake” OR “inhal$ mishandling” OR "incorrect inhaler use" OR "inhal$ ease of use" [as multipurpose terms] |
| AND | ”inhal$” or “autohaler” or “device$” or “metered dose inhal$” or “dry powder inhal$” or “breath-activated inhal$” or “spacer device” |
| **Search two (economic evaluations and health utility data)** | |
| “asthma” OR “chronic obstructive pulmonary disease” OR “COPD” [in abstract AND title] | |
| AND | *"cost"/ OR *"cost benefit analysis"/ OR *"cost control"/ OR *"cost effectiveness analysis"/ OR *"cost minimi$ation analysis"/ OR *"cost of illness"/ OR *"cost utility analysis"/ OR *economic evaluation/ OR *economics/ OR economics/ OR "Costs and Cost Analysis"/ OR "cost allocation"/ OR "cost control"/ OR "cost savings"/ [as MeSH Major Topic]  OR (standard gamble) OR (time trade off OR time tradeoff) OR (eq-5d or eq5d or euroquol*) OR (hui1 or hui2 or hui3) OR (sf6d OR sf 6d OR short form 6d OR shortform 6d OR sf sixd OR sf six d) OR (QALY OR quality-adjusted life year) [as multipurpose terms] |

Note: In this analysis, the term ‘critical error’ refers to both critical errors and critical steps that, when performed incorrectly, constitute critical errors. The grey literature search scanned proceedings from conferences between 2013 and 2016, namely by the International Society for Pharmacoeconomics and Outcomes Research, Association for Respiratory Care, European Respiratory Society, American Thoracic Society, and Respiratory Drug Delivery.

Table S2: Critical errors cited in the literature, stratified by device type

| **Device type** | **Critical errors reported** | **Number of studies which categorize activity as critical errors** |
| --- | --- | --- |
| pMDI [[1-25](#_ENREF_1)] |  | n=25 |
|  | Did not activate device correctly | 1† |
|  | Did not actuate once at beginning of inhalation (did not coordinate) | 19 |
|  | Actuation after end or before start of inhalation | 9 |
|  | Actuation against teeth, lips or tongue | 3 |
|  | Inadequate lip closure | 4 |
|  | No (or short) breath holding after inhalation | 12 |
|  | Not breathing slowly and deeply | 20 |
|  | Not breathing out gently | 1 |
|  | Failure to remove cap | 17 |
|  | Inhalation through nose | 4 |
|  | Not inhaling through mouthpiece, and no seal | 3 |
|  | Place MDI in mouth or 1—2 inches away from mouth | 3 |
|  | No forceful inhalation | 1 |
|  | Inhaler upside down (did not keep upright) | 6 |
|  | Did not shake inhaler well | 15 |
|  | Stopping inhalation as device is fired | 3 |
|  | No inhalation | 2 |
|  | Did not wait for at least 30 second before next actuation  Did not keep head (mostly) upright | 4  1 |
|  | Did not replace cap | 1 |
|  | Did not rinse mouth after dosing | 1 |
| pMDI with spacer  [[1](#_ENREF_1), [5](#_ENREF_5), [8](#_ENREF_8), [10-13](#_ENREF_10), [15](#_ENREF_15), [17](#_ENREF_17), [25](#_ENREF_25), [26](#_ENREF_26)] |  | n=11 |
|  | Did not activate device correctly | 1† |
|  | Did not shake device | 9 |
|  | Did not connect MDI to spacer | 4 |
|  | Did not place canister correctly in spacer | 3 |
|  | Removed spacer from mouth between breaths | 1 |
|  | Did not inhale at least three times, slowly | 5 |
|  | Actuate the device once (no more) | 7 |
|  | Did not remove cap | 5 |
|  | Did not hold inhaler upright | 1 |
|  | Did not form a seal over facemask with mouth | 5 |
|  | Did not take 5–6 deep breaths | 5 |
|  | Did not wait at least 30 seconds before next actuation | 2 |
|  | Did not coordinate activation and inhalation | 4 |
|  | Did not hold breath for at least 10 seconds | 3 |
|  | Did not remove spacer from mouth before exhalation | 2 |
|  | Did not remove inhaler from spacer | 1 |
|  | Did not replace cap after use | 2 |
| *Diskus*® DPI  [[1-4](#_ENREF_1), [7](#_ENREF_7), [8](#_ENREF_8), [12](#_ENREF_12), [18](#_ENREF_18), [19](#_ENREF_19), [27-33](#_ENREF_27)] |  | n=16 |
|  | Blowing into device before inhalation | 2 |
|  | Failed to open the device | 10 |
|  | Failure of loading | 6 |
|  | Device not level | 6 |
|  | Failure of priming | 6 |
|  | Gradual increase in speed on inhalation | 1 |
|  | Did not push the lever back fully (until it clicks, to prime the device) | 10 |
|  | Sliding back the lever after inhalation has started | 1 |
|  | Dropped device or held it with the open mouthpiece facing downwards | 2 |
|  | Exhaling into mouthpiece of device after loading | 3 |
|  | Mouthpiece between teeth and lips | 2 |
|  | Did not hold breath for at least 3s | 4 |
|  | Not exhaling away from mouthpiece | 5 |
|  | Not inhaling through the mouthpiece (i.e. inhaling through lever grooves) | 5 |
|  | Not sealing lips round mouthpiece during inhalation | 3 |
|  | Lips surround the mouthpiece shallowly against teeth or tongue | 5 |
|  | Inhaling by nose | 2 |
|  | Not activating inhaler | 1 |
|  | Not inhaling forcefully or deeply enough | 14 |
|  | Did not wait sufficient time before second use | 1 |
|  | Did not close cap after use | 1 |
|  | Failure to detect when device is empty | 1 |
| *Turbuhaler*® DPI  [[1-4](#_ENREF_1), [7](#_ENREF_7), [8](#_ENREF_8), [11](#_ENREF_11), [12](#_ENREF_12), [18](#_ENREF_18), [19](#_ENREF_19), [25-27](#_ENREF_25), [30](#_ENREF_30), [33](#_ENREF_33)] |  | n=15 |
|  | Blowing into device before inhalation | 1 |
|  | Did not lift off the cover | 3 |
|  | Did not remove cap from inhaler | 2 |
|  | Did not exhale away from mouthpiece | 3 |
|  | Failure of loading | 3 |
|  | Failure of priming | 5 |
|  | Did not hold the device upright (while priming) | 13 |
|  | Turn upright without occluding air vents | 6 |
|  | Not keeping the inhaler no more than 45 degrees from the vertical axis during loading | 5 |
|  | Did not rotate the grip fully (i.e. until a click was heard) | 3 |
|  | Did not rotate grip anti-clockwise and then back until a click is heard | 8 |
|  | Exhaling into mouthpiece of device after loading | 4 |
|  | Did not exhale to residual volume | 1 |
|  | Did not inhale through mouthpiece | 3 |
|  | Inhaling through nose | 6 |
|  | Not sealing lips round mouthpiece during inhalation | 7 |
|  | Not inhaling forcefully or deeply enough | 15 |
|  | Did not hold breath | 2 |
|  | Did not wait adequate time before second use/ did not complete steps a second time when extra dose required | 2 |
|  | Did not close cap/cover after use | 2 |
|  | Did not check dose counter and expiry date | 1 |
| *Genuair*® DPI[[34](#_ENREF_34), [35](#_ENREF_35)] |  | n=2 |
|  | Blows into inhaler when the dose is loaded | 1 |
|  | Button pressed whilst inhaling | 1 |
|  | Control window not green before inhalation | 2 |
|  | Did not exhale before introducing mouthpiece into mouth | 1 |
|  | Did not inhale sufficiently through the inhaler (control window does not change from green to red) | 1 |
|  | Does not hold the inhaler upright with green button facing upwards for priming (45 degree tolerance), as part of dose preparation | 1 |
|  | Exhaled into inhaler when dose is loaded | 1 |
|  | Inhalation immediately stopped upon hearing the click | 1 |
|  | Inhaler is put down with the control window showing green | 2 |
|  | Inhaler not held horizontally with green button facing upwards for priming the dose (45 degree tolerance) | 1 |
|  | Inhaler shaken with mouthpiece facing the ground after priming the dose | 1 |
|  | Mouth cap not removed before use | 2 |
|  | Mouthpiece facing downwards after preparation of dose | 1 |
|  | Shakes the inhaler with the mouthpiece facing the ground after priming, as part of dose preparation | 1 |
|  | Unable to open pouch with scissors | 1 |
| *Aerolizer*® DPI  [[2-4](#_ENREF_2), [7](#_ENREF_7), [13](#_ENREF_13), [18](#_ENREF_18), [27](#_ENREF_27)] |  | n=7 |
|  | Blowing in the device before inhalation | 1 |
|  | Did not open the dust cap and mouthpiece | 2 |
|  | Did not insert a capsule | 1 |
|  | Did not insert a capsule in the well and close | 2 |
|  | Failure of loading | 2 |
|  | Did not place the capsule in the appropriate chamber | 1 |
|  | Failure of priming | 2 |
|  | Did not close the mouthpiece so a click was heard | 1 |
|  | Did not pierce capsule | 2 |
|  | Did not press buttons to pierce the capsule and then release them | 2 |
|  | Did not press the lateral buttons of the inhaler | 1 |
|  | Exhaling into mouthpiece of device after loading | 2 |
|  | Did not exhale to FRC before inhalation | 1 |
|  | Not sealing lips round mouthpiece during inhalation | 4 |
|  | Did not keep device horizontal | 1 |
|  | Did not inhale through the mouthpiece | 2 |
|  | Inhaling by nose | 3 |
|  | Did not breathe in rapidly and deeply | 3 |
|  | Slow and not forceful inhalation | 3 |
|  | Did not exhale away from mouthpiece | 2 |
| *Handihaler*® DPI [[3](#_ENREF_3), [4](#_ENREF_4), [12](#_ENREF_12), [15](#_ENREF_15), [18](#_ENREF_18), [25-28](#_ENREF_25)] |  | n=9 |
|  | Did not open device correctly prior to priming | 3 |
|  | Did not place a capsule in the chamber | 4 |
|  | Failure of loading | 1 |
|  | Put a damaged capsule in the chamber | 1 |
|  | Did not close the mouthpiece until it clicks | 3 |
|  | Failure of priming | 2 |
|  | Did not pierce the capsule by pressing the blue button | 2 |
|  | Did not hold device upright as part of priming | 2 |
|  | Did not release the blue button before inhaling | 1 |
|  | Not releasing the pressed piercing button throughout inhalation | 1 |
|  | Shook the device with the mouthpiece facing the ground after priming | 2 |
|  | Did not exhale to FRC before placing mouthpiece in mouth | 2 |
|  | Exhaling into mouthpiece of device after loading | 1 |
|  | Not sealing lips around mouthpiece during inhalation | 5 |
|  | Inhaling by nose | 2 |
|  | Slow and not forceful inhalation | 6 |
|  | Did not hold breath for 10s | 3 |
|  | Did not remove inhaler from mouth whilst holding breath and exhale away from mouthpiece | 2 |
|  | Did not repeat inhalation step after first inhalation to ensure capsule is emptied | 2 |
|  | Does not make capsule rattle before putting the device down | 2 |
|  | Did not open lid and mouthpiece | 2 |
|  | Did not hold inhaler upright and press blue button fully | 1 |
|  | Blows into inhaler when the dose is loaded | 1 |
|  | Does not expire inhaled drug | 1 |
| *Diskhaler*® DPI [[1](#_ENREF_1), [8](#_ENREF_8)] |  | n=2 |
|  | Did not keep the device flat | 1 |
|  | Did not correctly load the device; lid was not raised | 1 |
|  | Did not inhale forcefully and deeply | 2 |
|  | Did not open and close device | 1 |
| *Elpenhaler*® [[28](#_ENREF_28), [32](#_ENREF_32)] |  | n=2 |
|  | Not closing mouthpiece correctly | 2 |
|  | Exhaling in mouthpiece | 2 |
|  | Not gently pulling off the protruding end of the[[1](#_ENREF_1)] strip | 2 |
|  | Mouthpiece not between teeth and lips | 2 |
|  | Not opening protective cap | 2 |
|  | Not placing blister strip correctly on supporting surface | 2 |
|  | Not pushing back mouthpiece to reveal supporting surface | 2 |
|  | Not taking blister strip from storage compartment | 2 |
|  | Did not Inhale | 2 |
| *Cyclohaler*® DPI [[1](#_ENREF_1), [8](#_ENREF_8)] |  | n=2 |
|  | Did not keep device upright during perforation | 1 |
|  | Did not correctly load the device by pressing the blue buttons once | 1 |
|  | Not inhaling forcefully and deeply | 2 |
|  | Not opening device in vertically position with body below | 1 |
|  | Not placing capsule in the device | 1 |
|  | Not pushing the buttons once | 1 |
|  | Not turning back mouthpiece | 1 |
| *Rotahaler*® [[8](#_ENREF_8), [23](#_ENREF_23)] |  | n=2 |
|  | Not correctly loading device for inhalation; not rotating the base | 2 |
|  | Not holding DPI in correct position; not keeping upright during perforation | 2 |
|  | Not Inhaling forcefully and deeply | 2 |
| *Breezhaler*® DPI [[34](#_ENREF_34)] |  | n=1 |
|  | Air entry blocked by finger whilst inhaling | 1 |
|  | Buttons not released during inhalation | 1 |
|  | Buttons on both sides not pressed simultaneously (capsule not pierced completely) | 1 |
|  | Buttons to pierce capsule pressed more than once | 1 |
|  | Capsule does not spin around in the chamber when inhaling due to possible problem with either the inhaler or the capsule | 1 |
|  | Capsule not removed after inhalation | 1 |
|  | Capsule placed directly into mouthpiece instead of into capsule chamber | 1 |
|  | Capsule swallowed instead of inhaling via inhaler | 1 |
|  | Damaged or contaminated capsule placed into capsule chamber | 1 |
|  | Did not exhale before introducing mouthpiece into mouth | 1 |
|  | Exhaled into inhaler (mouthpiece) when capsule is already placed into the chamber and mouthpiece is in patient's mouth | 1 |
|  | Inhalation not repeated due to powder residue in the capsule and capsule immediately removed without checking for existence of powder residue | 1 |
|  | Inhalation not sufficiently strong to hear the buzzing indicating that the capsule is spinning around the chamber | 1 |
|  | Inhaler not held upright after capsule was pierced | 1 |
|  | Inhaler not held with buttons facing left and right | 1 |
|  | Inhaler shaken after capsule has been pierced | 1 |
|  | Mouthpiece facing downwards after preparation of dose | 1 |
|  | Mouthpiece not closed correctly before piercing the capsule | 1 |
|  | Second capsule exposed to light in the process of opening first capsule and exposed capsule not discarded | 1 |
|  | Unable to open mouthpiece | 1 |
|  | Unable to pull cap off | 1 |
|  | Unable to remove capsule from the blister | 1 |
| *Easi-Breathe*® breath  actuated pMDI [[9](#_ENREF_9)] |  | n=1 |
|  | Not breathing in slowly and deeply | 1 |
|  | Not breathing out gently | 1 |
|  | Not holding inhaler upright | 1 |
|  | Inhaling through the nose | 1 |
|  | No inhalation | 1 |
|  | Put lever down before inhalation | 1 |
|  | Not putting mouthpiece in mouth and closing lips | 1 |
|  | Not raising the level to the vertical position | 1 |
|  | Not removing/ opening cap | 1 |
|  | Stop breathing at actuation | 1 |
| *Autohaler*®  breath-actuated [[5](#_ENREF_5), [9](#_ENREF_9)] |  | n=2 |
|  | Not breathing in slowly and deeply | 1 |
|  | Not breathing out gently | 1 |
|  | Not holding inhaler upright | 1 |
|  | Inhaling through the nose | 1 |
|  | No inhalation  Not pushing up top lever | 1 |
|  | Put lever down before inhalation | 1 |
|  | Not putting mouthpiece in mouth and closing lips | 1 |
|  | Not raising the lever to the vertical position  Not removing cap | 1  2 |
|  | Stop breathing at actuation | 1 |
| *Accuhaler*® [[11](#_ENREF_11), [15](#_ENREF_15), [25](#_ENREF_25), [26](#_ENREF_26)] |  | n=4 |
|  | Failed to identify if the product was within the expiration date | 1 |
|  | Not holding Accuhaler horizontally | 2 |
|  | Not pushing the lever away from the mouthpiece until you hear the ‘click’ | 5 |
|  | Did not breathe out to residual volume before inhalation | 1 |
|  | Did not create an adequate seal with lips around mouthpiece | 2 |
|  | Did not hold breath for correct length of time | 3 |
|  | Did not breathe out away from inhaler | 4 |
|  | Not Inhaling forcefully and deeply | 4 |
| *Revolizer*® DPI [[36](#_ENREF_36)] |  | n=1 |
|  | Did not hold the breath after inhalation | 1 |
|  | Inhalation was slow | 1 |
| *Pulvinal*® DPI [[13](#_ENREF_13)] |  | n=1 |
|  | Not rotating the inhaler body counter-clockwise | 1 |
|  | Not rotating the inhaler body clockwise until it clicks | 1 |
|  | Not inhaling quickly and deeply | 1 |
| *Fostex*® DPI [[22](#_ENREF_22)] |  | n=1 |
|  | Protective cap not removed | 1 |
|  | Mouthpiece not placed between lips | 1 |
|  | Did not inhale slowly while actuating the device | 1 |
| *Pulmojet*® DPI [[33](#_ENREF_33)] |  | n=1 |
|  | Did not remove cap | 1 |
|  | Placed fingers or mouth completely over holes in the device body | 1 |
|  | Fails to put in mouth and seal lips around mouthpiece | 1 |
|  | Inhalation is not fast enough to hear soft plop | 1 |
|  | Cap is not securely screwed down and no click is heard | 1 |
|  | Fails to inhale through mouthpiece | 1 |
| Critical errors listed are inclusive of stated critical errors and steps which, if not completed, were classed as critical errors. As wordings / terminology of the same critical errors differed between studies (n=299 critical errors in total), the table presents a consolidated list per device type.  ‘n’ refers to the total amount of studies reporting critical errors for corresponding device.  *: did not differentiate between the two devices.  †: this error was defined as not fulfilling the technical specifications of the device as recommended by the device manufacturer. | | |

Table S3: Studies which report link between control and resource use, economic burden and worsened QoL

| **Reference** | **Key conclusions** |
| --- | --- |
| Demoly *et al.* [[37](#_ENREF_37)] | “at least well-controlled asthma” was associated with:   - Improved SF-12 scores (physical components) - significantly lower number of healthcare contacts in the previous 6 months - significantly less impact on Work Productivity Loss and Activity Impairment |
| Doz *et al*. [[38](#_ENREF_38)] | EQ-5D scores were worse in patients with uncontrolled asthma and partially controlled asthma in comparison to those with controlled asthma (health utility score: 0.63–0.69, 0.78–0.82 and 0.88–0.89, respectively, p<0.0001)  Average cost (euros/3-months/patient) were higher for uncontrolled asthma than controlled asthma:  France (p<0.0001):   - €85.4 well controlled - €314.0 partially controlled - €537.9 poorly controlled   Spain (p<0.0001):   - €152.6 well controlled - €241.2 partially controlled - €556.8 poorly controlled |
| Szende *et al*. [[39](#_ENREF_39)] | QoL, as measured by the EQ-5D, SF-35 and SGRQ is worse in asthma patients with poor control in comparison to those with good control:  EQ-5D (high score is better):   - 0.93 good control - 0.52 poor control   SF-36 (high score is better):   - 48 physical, 55 mental good control - 31 physical, 44 mental poor control   SGRQ (low score is better)   - 21 good control - 60 poor control |
| Vervloet *et al*. [[40](#_ENREF_40)]] | Mean per patient annual costs were higher for not-well controlled patients:   - €232 (95%CI: €192–286) well controlled - €512 (€404–660) poorly controlled - €1,604 (€1,219–€2,084) not-well controlled |
| Williams *et al*. [[41](#_ENREF_41)] | Patients with controlled asthma reported (all p<0.001):   - Better QoL (higher SF-8 physical and mental scores) - fewer emergency room visits - fewer hospitalization days and medical provider visits - lower work productivity loss - reduced activity impairment |

| Table S4: Definitions of a critical error with a device | | |
| --- | --- | --- |
| **Authors** | **Year** | **Definition of critical errors** |
| ***Publications stating that a critical error would certainly affect inhalation and drug delivery:*** | | |
| Basheti *et al.* [[30](#_ENREF_30)] | 2011 | “Essential step: if not performed correctly, little or no medication will reach the lungs” |
| Batterink *et al.* [[12](#_ENREF_12)] | 2012 | “Critical errors were defined as those resulting in little or no medication reaching the lungs.” |
| Deerojanawong *et al.* [[10](#_ENREF_10)] | 2009 | “The MDI checklist contained 10 steps; 7 of which were considered essential for adequate drug delivery.” |
| Van der Palen  *et al.* [[28](#_ENREF_28)] | 2013 | “Critical errors were defined as those that will lead to an insufficient drug deposition in the lungs.” |
| Van der Palen  *et al.* [[35](#_ENREF_35)] | 2013 | “… critical errors included those that impeded delivery of sufficient dose or drug deposition in the lungs.” |
| Voshaar *et al.* [[42](#_ENREF_42)] | 2014 | “A critical error was defined as any error that prevented a dose being delivered.” |
| Wieshammer  *et al.* [[27](#_ENREF_27)] | 2008 | “… operating errors that make significant deposition of the medication to the lung impossible were defined *a priori* as ‘essential’.” |
| Capstick *et al.* [[43](#_ENREF_43)] | 2013 | “at least one critical error, significantly affecting lung deposition” |
| Nama *et al.* [[44](#_ENREF_44)] | 2014 | “critical errors that would result in inadequate amounts of drug reaching the lung“ |
| Pothirat *et al.* [[15](#_ENREF_15)] | 2016 | “…Essential steps required for adequate drug delivery” |
| Madkour *et al.* [[18](#_ENREF_18)] | 2015 | “Essential steps were defined as those considered necessary for optimal delivery of the active drug into the lungs” |
| Vanderman *et al.* [[14](#_ENREF_14)] | 2015 | “Errors that if made will significantly reduce or eliminate the delivery of medication to the lungs” |
| Chrystyn *et al.*[[33](#_ENREF_33)] | 2016 | “those that would definitely preclude adequate dose delivery to the lungs” |
| Ozturk *et al.* [[45](#_ENREF_45)] | 2015 | “…Causing insufficient drug inhalation…” |
| ***Publications stating that a critical error could affect inhalation and drug delivery:*** | | |
| Coelho *et al.* [[13](#_ENREF_13)] | 2011 | “… we identified the “key steps” in performing the inhalation maneuvers … the steps that, when incorrectly performed by users, can significantly affect total deposition of the dose in the lungs.” |
| Giraud *et al.* [[9](#_ENREF_9)] | 2011 | “… critical errors were defined, per Molimard *et al*., as errors that could substantially affect dose delivery to the lung.” |
| Hagmolen *et al.* [[8](#_ENREF_8)] | 2008 | “When errors are made regarding key actions, it is likely that no, or only an insignificant amount of medicine will be inhaled.” |
| Mehuys *et al.* [[5](#_ENREF_5)] | 2010 | “… crucial errors (i.e., errors probably leading to no drug deposition in the lungs)...” |
| Molimard *et al.* [[2](#_ENREF_2)] | 2008 | “A critical error is one that is likely to affect delivery of the full dose of the drug to the bronchi…” |
| Rajan *et al.* [[36](#_ENREF_36)] | 2014 | “The errors were classified as critical errors – those that can have a significant effect on the drug delivery to the lungs or non‑critical errors – those that do not affect dose delivery to the lungs.” |
| Rootmensen  *et al.* [[1](#_ENREF_1)] | 2010 | “When one or more errors were made regarding these essential steps, we considered it unlikely that a significant amount of medicine would be inhaled.” |
| Schulte *et al.* [[46](#_ENREF_46)] | 2008 | “Handling errors that could result in a reduced or zero dose delivery were defined as critical handling errors.” |
| Molimard *et al.* [[47](#_ENREF_47)] | 2005 | “Inhalation errors were considered critical if they could have substantially affected drug delivery to the lung” |
| Van der Palen *et al.* [[48](#_ENREF_48)] | 2012 | “critical errors that may result in receiving no drug or a suboptimal dose” |
| Westerik *et al.* [[31](#_ENREF_31)] | 2016 | “Serious inhaler technique errors identified by the HCPs were defined as errors potentially limiting drug uptake to the lungs” |
| Thomas *et al.* [[49](#_ENREF_49)] | 2015 | “Errors likely to result in significantly reduced drug delivery” |
| Chrystyn *et al.* [[50](#_ENREF_50)] | 2015 | “those potentially affective dose delivery” |
| ***Publications stating that a critical error would make aerosol therapy useless:*** | | |
| Li *et al.* [[29](#_ENREF_29)] | 2014 | “The analysis focused on the critical errors that are likely to make aerosol therapy useless...” |
| Melani *et al.* [[3](#_ENREF_3)] | 2011 | “… critical errors, which are likely to make therapy with aerosols useless...” |
| Melani *et al.* [[4](#_ENREF_4)] | 2012 | “… critical errors, which are likely to make therapy aerosol useless.” |
| Capanoglu *et al.* [[19](#_ENREF_19)] | 2015 | “steps that are essential for correct use of inhaler medications”* |
| ***Publication stating that a critical error would make aerosol therapy useless by affecting inhalation or drug delivery:*** | | |
| Pascual *et al.* [[34](#_ENREF_34)] | 2015 | “Critical errors were defined as those that compromised the potential benefit of the treatment, such as impeding drug deposition in the lungs or the delivery of an insufficient dose...” |
| Note: In this table the term “critical error” refers to both critical errors and critical steps that, when performed incorrectly, constitute critical errors. *The definition provided by Capanoglu et al. is considered to infer that making an error in an “essential” step will mean that the inhaler is used incorrectly, and that the benefit of treatment is compromised. | | |

# References

1. Rootmensen G, Van Keimpema AR, Jansen HM, De Haan RJ: **Predictors of incorrect inhalation technique in patients with asthma or COPD.** *American Journal of Respiratory and Critical Care Medicine* 2010, **181:**A3812.

2. Molimard M, Gros VL: **Impact of patient-related factors on asthma control.** *Journal of Asthma* 2008, **45:**109-113.

3. Melani AS, Bonavia M, Cilenti V, Cinti C, Lodi M, Martucci P, Serra M, Scichilone N, Sestini P, Aliani M, Neri M: **Inhaler mishandling remains common in real life and is associated with reduced disease control.** *Respiratory Medicine* 2011, **105:**930-938.

4. Melani AS, Canessa P, Coloretti I, Deangelis G, Detullio R, Del Donno M, Giacobbe R, Scarlato I, Serafini A, Barbato N, et al: **Inhaler mishandling is very common in patients with chronic airflow obstruction and long-term home nebuliser use.** *Respiratory Medicine* 2012, **106:**668-676.

5. Mehuys E, Boussery K, Adriaens E, van Bortel L, De Bolle L, van Tongelen I, Remon JP, Brusselle G: **COPD management in primary care: An observational, community pharmacy-based study.** *Annals of Pharmacotherapy* 2010, **44:**257-266.

6. Loh LC, Teng CL, Teh PN, Koh CN, Vijayasingham P, Thayaparan T: **Metered-dose inhaler technique in asthmatic patients - a revisit of the Malaysian scene.** *The Medical journal of Malaysia* 2004, **59:**335-341.

7. Khassawneh BY, Al-Ali MK, Alzoubi KH, Batarseh MZ, Al-Safi SA, Sharara AM, Alnasr HM: **Handling of inhaler devices in actual pulmonary practice: Metered-dose inhaler versus dry powder inhalers.** *Respiratory Care* 2008, **53:**324-328.

8. Hagmolen of ten Have W, van de Berg NJ, Bindels PJ, van Aalderen WM, van der Palen J: **Assessment of inhalation technique in children in general practice: increased risk of incorrect performance with new device.** *Journal of Asthma* 2008, **45:**67-71.

9. Giraud V, Allaert FA, Roche N: **Inhaler technique and asthma: Feasability and acceptability of training by pharmacists.** *Respiratory Medicine* 2011, **105:**1815-1822.

10. Deerojanawong J, Na Sakolnakorn VP, Prapphal N, Hanrutakorn C, Sritippayawan S: **Evaluation of metered-dose inhaler administration technique among asthmatic children and their caregivers in Thailand.** *Asian Pacific Journal of Allergy and Immunology* 2009, **27:**87-93.

11. Bryant L, Bang C, Chew C, Hee Baik S, Wiseman D: **Adequacy of inhaler technique used by people with asthma or chronic obstructive pulmonary disease.** *Journal of Primary Health Care* 2013, **5:**191-198.

12. Batterink J, Dahri K, Aulakh A, Rempel C: **Evaluation of the use of inhaled medications by hospital inpatients with chronic obstructive pulmonary disease.** *Canadian Journal of Hospital Pharmacy* 2012, **65:**111-118.

13. Coelho ACC, Souza-Machado A, Leite M, Almeida P, Castro L, Cruz CS, Stelmach R, Cruz AA: **Use of inhaler devices and asthma control in severe asthma patients at a referral center in the city of Salvador, Brazil.** *Jornal Brasileiro de Pneumologia* 2011, **37:**720-728.

14. Vanderman AJ, Moss JM, Bailey JC, Melnyk SD, Brown JN: **Inhaler misuse in an older adult population.** *The Consultant Pharmacist* 2015, **30:**92-100.

15. Pothirat C, Chaiwong W, Phetsuk N, Pisalthanapuna S, Chetsadaphan N, Choomuang W: **Evaluating inhaler use technique in COPD patients.** *The International Journal of Chronic Obstructive Pulmonary Disease* 2015, **10:**1291-1298.

16. Manriquez P, Acuna AM, Munoz L, Reyes A: **Study of inhaler technique in asthma patients: differences between pediatric and adult patients.** *Jornal Brasileiro de Pneumologia* 2015, **41:**405-409.

17. Maricoto T, Rodrigues LV, Teixeira G, Valente C, Andrade L, Saraiva A: **Assessment of inhalation technique in clinical and functional control of asthma and chronic obstructive pulmonary disease.** *Acta Medica Portuguesa* 2015, **28:**702-707.

18. Madkour A, Galal I: **Do Egyptian patients use their inhalers correctly? A checklist auditing for inhalation devices usage techniques.** *Egyptian Journal of Chest Diseases and Tuberculosis* 2015, **64:**497-504.

19. Capanoglu M, Dibek Misirlioglu E, Toyran M, Civelek E, Kocabas CN: **Evaluation of inhaler technique, adherence to therapy and their effect on disease control among children with asthma using metered dose or dry powder inhalers.** *Journal of Asthma* 2015, **52:**838-845.

20. Dalcin PTR, Grutcki DM, Laporte PP, de Lima PB, Menegotto SM, Pereira RP: **Factors related to the incorrect use of inhalers by asthma patients.** *Jornal Brasileiro de Pneumologia* 2014, **40:**13-20.

21. Elgendy MO, Abdelrahim ME, Eldin RS: **Potential benefit of repeated MDI inhalation technique counselling for patients with asthma.** *European Journal of Hospital Pharmacy* 2015, **22:**318-322.

22. Kuna P, Kupryś-Lipińska I, Dębowski T: **Control of asthma in adults treated with beclomethasone and formoterol in extrafine particle formulation in a real-life setting in Poland: the CASPER noninterventional, observational trial.** *Polskie Archiwum Medycyny Wewnetrznej* 2015, **125:**731-740.

23. Sangita P, Gharti KP, Laxman B: **Assessment of inhalation techniques in COPD patients using metered-dose inhaler and Rotahaler at a tertiary care hospital in Nepal.** *International Research Journal of Pharmacy* 2015, **6:**288-293.

24. Udwadia ZZ, Kathar S, Shah HD, Pandey K, Rastogi S, Mullerpattan J: **Who will teach the teachers: an analysis of the inhaler technique of Indian patients and health care providers.** *American Journal of Respiratory & Critical Care Medicine* 2013**:**A-5026.

25. Williams J, Mault S, Garner N, Burhan H, Zaidi S: **Breaking down bad inhaler technique: Device specific advice.** *American Journal of Respiratory and Critical Care Medicine* 2012, **185:**A3332.

26. Sriram KB, Percival M: **Suboptimal inhaler medication adherence and incorrect technique are common among chronic obstructive pulmonary disease patients.** *Chronic respiratory disease* 2015, **13:**13-22.

27. Wieshammer S, Dreyhaupt J: **Dry powder inhalers: Which factors determine the frequency of handling errors?** *Respiration* 2008, **75:**18-25.

28. Van Der Palen J, Van Der Valk P, Goosens M, Groothuis-Oudshoorn K, Brusse-Keizer M: **A randomised cross-over trial investigating the ease of use and preference of two dry powder inhalers in patients with asthma or chronic obstructive pulmonary disease.** *Expert Opinion on Drug Delivery* 2013, **10:**1171-1178.

29. Li H, Chen Y, Zhang Z, Dong X, Zhang G, Zhang H: **Handling of diskus dry powder inhaler in Chinese chronic obstructive pulmonary disease patients.** *Journal of Aerosol Medicine and Pulmonary Drug Delivery* 2014, **27:**219-227.

30. Basheti IAQ, E. Bosnic-Anticevich, S. Z. Armour, C. L. Khater, S. Omar, M. Reddel, H. K.: **User error with diskus and turbuhaler by asthma patients and pharmacists in Jordan and Australia.** *Respiratory Care* 2011, **56:**1916-1923.

31. Westerik JA, Carter V, Chrystyn H, Burden A, Thompson SL, Ryan D, Gruffydd-Jones K, Haughney J, Roche N, Lavorini F, et al: **Characteristics of patients making serious inhaler errors with a dry powder inhaler and association with asthma-related events in a primary care setting.** *Journal of Asthma* 2016, **53:**321-329.

32. Van Der Valk P, Goosens M, Groothuis-Oudshoorn K, Brusse-Keizer M, van der Palen J: **Comparing the ease of use, preference and satisfaction of Accuhaler/Diskus and Elpenhaler in patients with asthma or chronic obstructive pulmonary disease.** In *B15 What works? Determining the most effective treatments for respiratory diseases.* American Thoracic Society; 2013: A-2336

33. Chrystyn H, Price DB, Molimard M, Haughney J, Bosnic-Anticevich S, Lavorini F, Efthimiou J, Shan D, Sims E, Burden A: **Comparison of serious inhaler technique errors made by device-naïve patients using three different dry powder inhalers: a randomised, crossover, open-label study.** *BMC Pulmonary Medicine* 2016, **16:**12.

34. Pascual S, Feimer J, De Soyza A, Sauleda Roig J, Haughney J, Padulles L, Seoane B, Rekeda L, Ribera A, Chrystyn H: **Preference, satisfaction and critical errors with Genuair and Breezhaler inhalers in patients with COPD: a randomised, cross-over, multicentre study.** *NPJ Primary Care Respiratory Medicine* 2015, **25:**15018.

35. Van Der Palen J, Ginko T, Kroker A, Van Der Valk P, Goosens M, Padulles L, Seoane B, Rekeda L, Garcia Gil E: **Preference, satisfaction and errors with two dry powder inhalers in patients with COPD.** *Expert Opinion on Drug Delivery* 2013, **10:**1023-1031.

36. Rajan S, Gogtay J: **Ease-of-use, preference, confidence, and satisfaction with Revolizer , a novel dry powder inhaler, in an Indian population.** *Lung India* 2014, **31:**366-374.

37. Demoly P, Annunziata K, Gubba E, Adamek L: **Repeated cross-sectional survey of patient-reported asthma control in Europe in the past 5 years.** *European Respiratory Review* 2012, **21:**66-74.

38. Doz M, Chouaid C, Com-Ruelle L, Calvo E, Brosa M, Robert J, Decuypere L, Pribil C, Huerta A, Detournay B: **The association between asthma control, health care costs, and quality of life in France and Spain.** *BMC Pulmonary Medicine* 2013, **13:**15.

39. Szende A, Svensson K, Ståhl E, Meszaros A, Berta GY: **Psychometric and utility-based measures of health status of asthmatic patients with different disease control level.** *Pharmacoeconomics* 2004, **22:**537-547.

40. Vervloet D, Williams A, Lloyd A, Clark T: **Costs of managing asthma as defined by a derived Asthma Control TestTM score in seven European countries.** *European Respiratory Review* 2006, **15:**17-23.

41. Williams SA, Wagner S, Kannan H, Bolge SC: **The association between asthma control and health care utilization, work productivity loss and health-related quality of life.** *Journal of Occupational and Environmental Medicine* 2009, **51:**780-785.

42. Voshaar T, Spinola M, Linnane P, Campanini A, Lock D, Lafratta A, Scuri M, Ronca B, Melani AS: **Comparing usability of NEXThaler with other inhaled corticosteroid/long-acting beta2-agonist fixed combination dry powder inhalers in asthma patients.** *Journal of Aerosol Medicine and Pulmonary Drug Delivery* 2014, **27:**363-370.

43. Capstick T, Clifton I, Morgan J, Silcock J, Blenkinsopp A: **Inhaler technique: An unmet need in patients with difficult asthma?** *European Respiratory Journal* 2013, **42:**A-4893.

44. Nama S, Percival M, Harris M, Nolan-Neylan S, Sivakumaran P, Sriram K, Harland K: **Evaluation of inhaler technique and medication adherence in COPD patients.** *Respirology* 2014, **19:**A-TP146.

45. Ozturk C, Aldag Y: **Evaluation and importance of different types of inhaler device use in patients with chronic obstructive lung disease (COPD).** In *Respirology*. 2015: A-471.

46. Schulte M, Osseiran K, Betz R, Wencker M, Brand P, Meyer T, Haidl P: **Handling of and preferences for available dry powder inhaler systems by patients with asthma and COPD.** *Journal of Aerosol Medicine and Pulmonary Drug Delivery* 2008, **21:**321-328.

47. Molimard M: **How to achieve good compliance and adherence with inhalation therapy.** *Current Medical Research and Opinion, Supplement* 2005, **21:**S33-37.

48. Van Der Palen J, Ginko T, Kroker A, Van Der Valk P, Goosens M, Padulles L, Seoane B, Rekeda L, Gil EG: **Comparison of the preference, satisfaction, and critical errors made with Genuair and Handihaler in patients with COPD.** In *Chest*; *October*. 2012: A-717A.

49. Thomas M, Van der Palen J, Chrystyn H, Sharma R, Imber V, Zhu C-Q, Barnes N: **Inhaler errors and preference: a comparison of two multi-dose powder asthma inhalers.** In *Respirology*. 2015: A-573.

50. Chrystyn H, Bosnic-Anticevich S, Roche N, Molimard M, Haughney J, Lavorini F, Shan D, Sims E, Burden A, Ashton VL: **Prevalence of serious post-training inhaler technique errors made by device-naïve patients using three different dry powder inhalers (DPIs).** *European Respiratory Journal* 2015, **46:**A-P3927.
